# Insurance and Assurance

**Published:** 1893-12

**Authors:** 


### INSURANCE AND ASSURANCE.

The words "insurance" and "assurance" are used as synonyms in many instances, the primary meaning being the same. But, like many other words in the very accommodating and elastic English language, the latter has a second, even a third, and a fourth definition and meaning, strangely unlike the primary. We use it here as defined by Webster under the fourth head, "excess of boldness, impudence, audacity."

If certain insurance companies do not understand the words as synonyms—they are, at least, in the habit of associating them very closely. It may be that they—the insurance company officers—look at the matter in a different light from that which falls upon our optic; if so, we would be glad to have a statement of their position and views.

We refer to a custom,—for, the doing of the thing has become so frequent of late that it may be said to have become a custom,—with, however, only certain and a certain class of insurance concerns, of addressing a letter of inquiry to a physician—any physician, even a total stranger to them, who has in any way acquired any prominence in the profession—as to the qualifications of physicians who apply for, or whom they think of appointing to the position of medical examiner for their company. They seek this information, of course, in their own interest—for their own security and protection. They not only ask what one knows of the professional qualifications of the applicant, but want an opinion of his general fitness.

The thing can be best understood by giving in full the contents of the last letter of the kind we received,—and we sometimes get two or three in a day. We assume that the information thus sought is, or would be, valuable to the company making the inquiry, else they would not make it; and if it is valuable, the company should pay for it. Here is the form of inquiry generally used:

MEDICAL DEPARTMENT<br>

——— LIFE INSURANCE COMPANY,<br>

———, Nov. 27, 1893.

*F. E. Daniel, M. D., Austin, Texas:*

DEAR SIR:—Dr.———, of ———, Texas, who is an applicant for the position of medical examiner of the ——— Life Insurance Company, refers us to you as to his standing in his community, and we therefore request you to answer the questions asked below.

When you have written the answers, please inclose this paper in the within envelope. Any remarks you may have to make additional to what you state below, we shall be glad to have you write on the other side of this sheet, and whatever you may write will be regarded as entirely confidential.

Very respectfully,

———, M. D.,<br>

———. M. D., } Medical Directors.

1st.—Is he regarded as among the intelligent physicians of his locality?

Answer:.......................................

2d.—Do you think him as fit as any other physican in his locality for the position of Medical Examiner?

Answer:.......................................

3d.—Please state whether his standing for truthfulness and the carefnl, conscientious performance of every duty he undertakes is unquestioned among those who know him?

Answer:.......................................

4th.—Is his medical education sufficient for the duties of a Medical Examiner of a Life Insurance Company? And, *particularly*, we wish to know: Is he competent to make a scientific examination of the Chest by auscultation and percussion?

Answer: ... .............................. ............

.... ........................................................

I am a graduate of...........................College.

[Sign here] ——— ————M. D.

---

Note the wording of the modest request.

We do not know exactly how the company making the above request would define it, but it looks to us as covering the ground of Webster's fourth definition to a "t."

We are pleased to say that there are companies who recognize the value of such service, and pay for it. The Manhattan Life Insurance Company of New York, pays a stipulated fee for the nomination of each medical examiner; and the Hartford, Conn., of which Dr. C. D. Alton is the director, encloses a check every time he asks for information concerning a medical examiner or an appointment. Other companies have and pay a medical referee in each State.

An editorial on this subject appeared in the *Texas Sanitarian* for March, which so fully expresses our own views that we reproduce it in this issue. If there are two sides to the question we should be pleased to hear the other; to us it looks like a great imposition on good natured doctors.

It is not that it is very little trouble; to write a prescription, is very little trouble also; but it is because you are rendering a valuable service to a company well able to pay for it. In case you can thoroughly indorse the applicant you would have not so much hesitation in complying with the request, but suppose you do not know enough of his qualifications and general character to justify an indorsement? The temptation to any but a very conscientious man is very great to say "yes" to all such inquiries referred to him,—he has no interest in it, is not paid, and does not care to incur the ill will of applicant (which he would surely do) by refusing to indorse him. Below is the article from the *Texas Sanitarian:*

"BEATING THE DOCTOR.—There is a very prevalent custom on the part of life insurance companies of addressing letters of inquiry to physicians as to the habits, qualifications, moral, social and professional standing of those physicians who apply for, or to whom they contemplate offering an appointment as medical examiner. At first this was an exceptional occurrence; now it has become a universal custom. And, no longer content with detailed answers to interrogatories relating to the above, these insurance companies now ask a physician—a stranger, and totally disinterested—to send them minute information as to the eye-sight and hearing, the mental status, the integrity and honesty of the proposed examiner, together with information as to his pecuniary responsibility—if he pays his debts; and an *opinion* as to whether or not the candidate has *firmness* and *honesty* enough to resist—not exactly *bribery*, in expressed words, but "such influence as might be brought to bear" in the interest of the agent or the person to be examined. This modest request is accompanied by an assurance that the information thus sought to be obtained, if adverse to the applicant for appointment, 'will be held as confidential;' and a stamped envelope is enclosed for a reply.

"There are said to be two sides to every question; but for the life of us, we are unable to see but one side of this question, and that is, that the physician is being used to further the interest of these rich corporations, without even a hint of remuneration, or a bare 'thank ye,' Here the insurance company asks,—and we have never heard of but one refusal,—and always gets something for nothing. That 'something' is a valuable consideration, and ought, by every claim of right, justice and equity to be paid for.

"There are companies who recognize the value of the service thus rendered, and pay a stipulated fee for each recommendation, or information which will guide their medical director in selecting a medical examiner. These companies have, usually, a medical referee, to whom they apply for information, and they pay him from three to five dollars for each appointment made upon his recommendation. We are not speaking of this, the better class of insurance companies. But the majority of insurance concerns will propound the above list of questions to any prominent physician whose opinion may be thought desirable.

"The medical examiner protects the company from loss by a judicious selection of risks, and is paid $5 for his opinion; the physician who furnishes the information upon which the selection of a medical examiner is made no less protects the company from loss, and he is accorded a stamped envelope (for reply) for his pains. Not only this, but, as stated, in the majority of cases his opinion is asked,—not only what he *knows* of the party's habits and qualifications, etc., but an opinion of his general fitness. A physician's opinion is said to be his 'stock in trade,'

and is worth a money consideration; it has been so held by the courts. What right, then, have these rich concerns to ask it gratuitously?

"Not only that, but whatever responsibility may attach to an appointment of a medical examiner for an insurance company, is shifted from the shoulders of the medical director, who is paid in many instances ten and twenty thousand dollars salary, to the good-natured country or village doctor, who in most cases cheerfully submits to the imposition. There it is, in black and white, and will stand a record against him forever. Doctors are not infallible, and sometimes make mistakes. A favorable opinion may be given of an applicant who turns out to be unreliable, or mayhaps dishonest or incompetent. The medical director, with his fat salary, excuses himself, and throws the onus on his correspondent, to whom perhaps reference has been made by the applicant, and who has hastened to oblige the company—free of charge—under the vague notion, perhaps, that he is doing a brother physician a favor to recommend him.

"Now, this is the only thing that can be said in extenuation of the custom: Dr. A wishes to examine for a certain company; the occasional $3 or $5 fee will be, to him, an item. He makes application for the appointment, and is forthwith requested to funrish reference. He refers to Dr. B, who edits a journal or holds an office which mayhaps has given him some prominence. It may be that Dr. B knows Dr. A well, and knows him to be eminently trustworthy, and in every way fitted for the position. It is but just to Dr. A, then, that when interrogated as to his qualifications, Dr. B should say so. Or, it may be that Dr. B is barely acquainted, but on friendly terms, with Dr. A, and has no knowledge whatever of his capacities or character more than from general hearsay. Here, then, Dr. B is placed in a delicate and embarrassing position. Should he answer the medical director's interrogatories about Dr. A in such a way as to decide the director not to make the appointment, Dr. A immediately infers that Dr. B has not indorsed him. A breech of their pleasant relations follows; Dr. B has lost a friend, perhaps. In either case, Dr. B has rendered the insurance company an important service, one which will save them money, and he ought to be paid for it. True, in the first instance, he has rendered his friend a service; but he has rendered the insurance company a service none the less.

"The *Texas Sanitarian* here and now enters a protest against such an imposition. Just so long as the physicians who are called upon in this way will respond, just so long will the imposition be kept up. Let every physician who receives one of these modest requests comply with it faithfully, promptly, conscientiously, just as he does with all other demands upon his professional services; but let him also enclose his bill for the service, and we venture it will soon put a stop to the practice, or at least to the attempt to get *something* for *nothing*."—*Ed. in Texas Sanitarian.*

